# Tumour-based DNA methylation markers of breast cancer survival: a pooled analysis of 2157 cases

**DOI:** 10.1186/s13058-026-02327-3

**Published:** 2026-06-26

**Authors:** Elaheh Zarean, Shuai Li, Enes Makalic, Roger L. Milne, Graham G. Giles, Catriona McLean, Melissa C. Southey, Pierre-Antoine Dugué

**Affiliations:** 1 Precision Medicine, School of Clinical Sciences at Monash Health, Monash University, 246 Clayton Road, Clayton, VIC Australia; 2 Centre for Epidemiology and Biostatistics, Melbourne School of Population and Global Health, The University of Melbourne, Parkville, VIC Australia; 3 Department of Data Science and AI, Faculty of Information Technology, Monash University, Clayton, VIC Australia; 4 Cancer Epidemiology Division, Cancer Council Victoria, Melbourne, VIC Australia; 5 Anatomical Pathology, Alfred Health, The Alfred Hospital, Melbourne, VIC Australia

**Keywords:** DNA methylation, Breast cancer, Epigenome-wide association study, Survival, Prediction model

## Abstract

**Background:**

Tumour DNA methylation is a potentially valuable marker of breast cancer survival, but previous studies have been limited by relatively small sample sizes. This study aimed to use a large sample size to identify survival-associated DNA methylation markers and develop a methylation-based signature predictive of breast cancer survival.

**Methods:**

We used DNA methylation data from 2,157 breast tumours collected in the Melbourne Collaborative Cohort Study (MCCS) and nine publicly available datasets. An epigenome-wide association study (EWAS) was conducted for five-year overall survival (*N* = 1,992 cases). Subgroup analyses were carried out by estrogen receptor status. Pathway enrichment analyses were conducted to identify related biological pathways. Elastic net Cox regression was used to develop a signature of survival from DNA methylation data and clinical characteristics, trained on publicly available datasets and tested in the MCCS (*N* = 425) in addition to the main clinical characteristics. A set of triple-negative tumours (*N* = 165) with disease-free survival as the outcome was used for additional replication.

**Results:**

We identified 2,535 CpGs showing an association (*P *< 1 × 10^− 7^) with survival in age-adjusted models, including 281 after adjustment for estrogen receptor (ER) status. In ER-positive tumours, there were 389 associations, of which 315 were absent from the primary EWAS. A 228-CpG signature showed a strong association with survival in the MCCS after adjustment for clinical characteristics: per SD, HR = 1.7, 95%CI: 1.2–2.3, *P* = 0.001, with a C-index of 0.79, compared with a C-index of 0.75 without methylation information (C-index increase: 0.04, 95%CI: 0.01–0.10). The signature was also predictive of disease-free survival beyond clinical characteristics in triple-negative tumours (HR per SD = 1.4, 95% CI: 1.1–1.8).

**Conclusion:**

Our findings revealed numerous CpG sites where tumour DNA methylation was associated with breast cancer survival. A substantial improvement in the accuracy of five-year overall survival prediction was achieved by adding a 228-CpG epigenetic score to the main clinicopathological variables. These results suggest that DNA methylation markers may provide additional prognostic information beyond established clinicopathological factors.

**Supplementary Information:**

The online version contains supplementary material available at https://doi.org/10.1186/s13058-026-02327-3.

## Introduction

 Epigenetic alterations have been extensively studied as potential markers to unravel the complex heterogeneity of breast cancer [1]. Among these, DNA methylation, a well-established hallmark of cancer [2], plays important roles in cancer progression by modulating essential processes such as cellular proliferation, growth, and DNA repair [3–5], and offers molecular insights that extend beyond traditional clinicopathologic characteristics [6, 7].

The association between individual tumour DNA methylation markers and breast cancer survival has been investigated in previous studies, suggesting potential links, although with limited replication [8–12]. The Cancer Genome Atlas (TCGA) dataset has been used as a primary resource in many studies [8–10, 12–15], but the limited availability of comparable external datasets restricts the generalisability of findings. For example, De Almeida et al. [8] analysed data from 781 TCGA breast tumours and identified 386 CpGs that were differentially methylated in tumours compared with normal tissues. Several of these were nominally associated with overall survival, including three in genes that were not reported in the context of cancer before (*TDRD10*, *PRAC2*, and *TMEM132C*), with stronger associations observed for estrogen receptor (ER)-positive tumours [16]. Another study examined 692 breast tumours from TCGA and identified 271 differentially methylated CpGs, of which 15 were associated with survival (*P*<1 × 10^− 7^) across subgroup analyses (luminal A and luminal B), but these were not replicated in a Gene Expression Omnibus (GEO) dataset (GSE72308; 180 tumours) [9]. In a study of 158 triple-negative breast cancers (TNBC) from TCGA, DNA methylation at 86 CpGs was reported to be associated with survival, albeit using a non-stringent statistical significance threshold (*P* < 0.01). Using data from the Melbourne Collaborative Cohort Study (MCCS), we recently assessed associations of methylation markers with survival for CpGs previously identified in TCGA, and found that most signals were in the same direction, with about half reaching nominal statistical significance [17].

Other studies have used machine learning methods to derive DNA methylation-based signatures that best predicted survival outcomes [10–14, 18, 19], most of which were developed using TCGA as the primary training dataset and were less comprehensive in their inclusion of publicly available datasets. These generally reported CpG-based risk scores that stratified patients into prognostic groups, although with validation often restricted to TCGA-based subsets or small independent validation cohorts not used in model development. To our knowledge no attempts of external replication were conducted except our MCCS study, which found a varying degree of replication of these signatures [17], this reliance on a single dataset and limited external validation may have constrained the generalisability of these findings. Therefore, there has been a relative lack of large, well-powered studies with tumour DNA methylation and outcome follow-up available, which has hindered the discovery of consistent epigenetic markers of breast cancer survival.

In this study, we sought to enhance the identification of DNA methylation markers of breast cancer survival, by pooling tumour DNA methylation datasets with available survival data, including cases from the MCCS, TCGA, and eight publicly available datasets. We aimed to (i) carry out an epigenome-wide association study (EWAS) of breast cancer survival to identify relevant genes and their related pathways, and (ii) develop a signature of breast cancer survival and assess its predictive capacity beyond the main clinicopathological variables.

## Methods

### Study sample

**Fig. 1:**
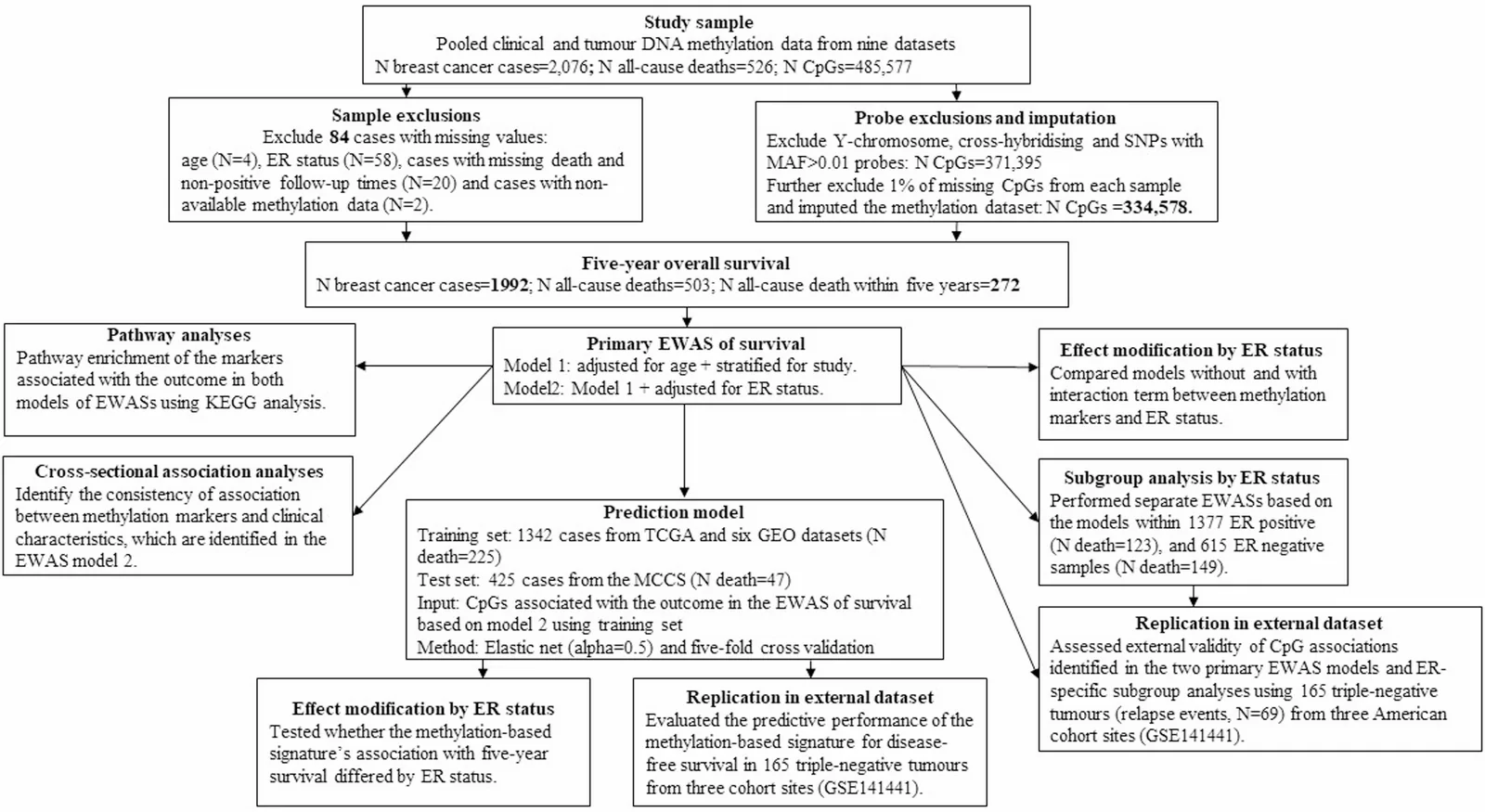
Flowchart of participant selection and analysis plan

Publicly available breast cancer tumour DNA methylation datasets were identified through a search of the published literature and the Gene Expression Omnibus (GEO) database, in addition to TCGA data. Studies were eligible for inclusion if genome-wide DNA methylation was profiled in tumour tissue using the Illumina HumanMethylation450 BeadChip (HM450K) assay and mortality follow-up data were available. No minimum sample size was required; however, datasets were required to provide processed beta values with standard quality control applied and sufficient clinical annotation (age at diagnosis, survival time, and vital status) to allow harmonisation across studies. Methylation datasets generated using other measurement platforms were not included. Using these criteria, nine datasets were included: the MCCS [17, 20] and eight publicly available datasets: TCGA [21] and from Gene Expression Omnibus (GEO): Jules Bordet Institute I (GSE72245), Jules Bordet Institute II (GSE72251), Trial of Principle (TOP) study (GSE72254), Baltimore hospitals (GSE37754), a German cohort (GSE69914), Australian Breast Cancer Tissue Bank (GSE78754), and Swedish and Icelandic cohorts (GSE75067). A summary of the clinical characteristics of the participants available for analysis after exclusions and methylation quality controls (flowchart Fig. 1) is provided in Table 1. Observations with missing values for age (*N* = 4) and ER status (*N* = 58) were excluded, as were 20 cases with missing or invalid follow-up times (e.g., follow-up time ≤ 0) and those with no DNA methylation data (*N* = 2), resulting in 1,992 cases with available information on five-year overall survival (N deaths = 272). Additionally, a set of 165 triple-negative tumours from three American cohorts with information on disease-free survival and available DNA methylation (HM450K, GSE141441) was used as a replication set. A description of each dataset is provided in Supplementary file.

**Table 1:** Summary characteristics of the nine studies (*N* = 1992) with tumour DNA methylation and survival information included in the analysis

		MCCS	TCGA	Jules Bordet Institute I	Jules Bordet Institute II	TOP study	Baltimore hospitals	Germany	The Australian breast cancer tissue bank	Swedish and icelandic cohorts	Total
Cases, *N*		425	688	116	117	51	59	302	63 TNBCs	171	1992
Age (years), Median (IQR)		64 (57–70)	58 (48–67)	54 (48–65)	59 (48–68)	50 (41.5–55)	51 (42.5–62.5)	61 (51–69)	52 (42–62)	49 (43–60)	
ER status, N (%)	Negative	84 (19.8)	159 (23.1)	53 (45.7)	49 (41.9)	51 (100)	31 (52.5)	48 (15.9)	63 (100)	77 (45)	615
	Positive	341 (80.2)	529 (76.9)	63 (54.3)	68 (58.1)		28 (47.4)	254 (84.1)		94 (55)	1377
IHC-based subtype N (%)	Luminal A	242 (56.9)	288 (41.9)	24 (20.7)	27 (23.1)			190 (62.9)			771
	Luminal B	87 (20.5)	62 (9)	32 (27.6)	29 (24.8)			31 (10.3)			241
	HER2	31 (7.3)	14 (2)	29 (25)	25 (21.4)			10 (3.3)			109
	TNBC/basal	65 (15.3)	78 (11.3)	31 (26.7)	34 (29.1)		14 (23.7)	30 (9.9)			315
	Missing	0 (0)	246 (35.8)	0 (0)	2 (1.7)		45 (76.3)	41 (13.6)			334
Grade, N (%)	1	89 (20.9)		24 (20.7)	28 (23.9)	1 (2)	7 (11.9)	41 (13.6)		20 (11.7)	210
	2	174 (40.9)		8 (6.9)	5 (4.3)	8 (15.7)	16 (27.1)	151 (50)		48 (28.1)	410
	3	140 (32.9)		84 (72.4)	84 (71.8)	42 (82.4)	28 (47.5)	109 (36.1)		86 (50.3)	573
	Missing	22 (5.2)		0 (0)	0 (0)	0 (0)	8 (13.6)	1 (0.3)		17 (9.9)	799
Stage, N (%)	I	246 (57.9)	116 (16.9)				5 (8.5)				367
	II	135 (31.8)	379 (55.1)				37 (62.7)				551
	III	44 (10.4)	187 (27.2)				0 (0)				231
	Missing	0	6 (0.9)				17 (28.8)				843
Tumour size (cm), median (IQR)		1.5 (1-2.2)		2.3 (1.7-3)	1.8 (1.4–2.5)			2 (1–2)			
	Missing	4 (0.9)		7 (5.9)	0 (0)			6 (2)			1049
All-cause deaths, N (%)		168 (39.5)	90 (13.1)	31 (26.7)	11 (9.4)	8 (15.7)	26 (44.1)	39 (12.9)	43 (68.3)	87 (50.9)	503
Follow-up time (year), median (IQR)		15.5 (11.9–19.5)	2.4 (1.4–4.9)	6.6 (5.3–8.3)	4.3 (3-5.5)	2.7 (2-3.8)	4.2 (2.2–7.3)	6.6 (5.4–9.1)	3.8 (2.4–6.3)	13.3 (3.8–21.3)	
5-year all-cause deaths, N (%)		47 (11.1)	63 (9.2)	21 (18.1)	9 (7.7)	7 (13.7)	23 (39)	24 (7.9)	29 (46)	49 (28.7)	272

IQR, interquartile range; ER, estrogen receptor; IHC, immunohistochemistry; MCCS, melbourne collaborative cohort study; TCGA, the cancer genome atlas; TOP study, trial of principal study; TNBC, triple-negative breast cancer

## Data pre-processing, normalisation and filtering

For five studies with raw data available (iDAT files; MCCS, TCGA, GSE72245, GSE72251 and GSE72254), normalisation and generation of methylation beta values was carried out using the *ENmix* pipeline [22], which includes background correction, dye-bias correction, and probe-type bias correlation, and has excellent reported performance [23, 24]. For the five remaining datasets (GSE37754, GSE69914, GSE78754, GSE75067 and GSE141441), we used the normalised data available at GEO (Supplementary file).

A series of filtering steps were implemented to ensure DNA methylation data quality. First, we excluded 416 probes annotated to the Y chromosome. Second, 38,941 potential cross-hybridizing probes, as identified by Chen et al. [25] and Benton et al. [26] were removed using the “*maxprobes”* R package. We also excluded 85,369 probes located within 10 base pairs of a known single nucleotide polymorphism (SNP) with minor allele frequency (MAF) greater than 0.01, as identified by Chen et al. [25] and Zhou et al. [27]. Of the remaining 371,395 CpGs, we further excluded those with > 1% missing values across samples in the pooled dataset. The remaining missing values were imputed using the k-nearest neighbours algorithm (k = 10) [28]. For all CpGs, methylation beta-values were standardised to a mean of 0 and standard deviation (SD) of 1 across samples in the pooled dataset.

## Survival outcome

We chose five-year overall survival as our primary endpoint as being more clinically relevant and available in all studies, yielding a total of 272 deaths from any cause within five years among 1,992 patients. Only three studies (MCCS, GSE37754, and GSE78754) had available cause-specific survival information (e.g., breast cancer-specific deaths, *N* = 119). GSE141441 only had information on disease-free survival (*N* = 69 events) and was used for replication.

### Statistical analysis

#### Epigenome-wide association studies

An EWAS of five-year overall survival (*N* = 1,992; N five-year all-cause death = 272) was conducted using Cox proportional hazards regression models. Two models were used: (i) Model 1 adjusted for age and stratified by study, allowing for study-specific baseline hazards while assuming a common hazard ratio (HR) across studies [29], and (ii) Model 2 additionally adjusted for ER status. Effect modification by ER status was assessed using likelihood ratio tests comparing models with and without an interaction term between methylation markers and ER status. Subgroup analyses were also conducted by ER status (ER-positive: N cases = 1,377, N deaths = 123 and ER-negative: N cases = 615, N deaths = 149). A p-value threshold of *P *< 1 × 10^− 7^, which approximately corresponds to the Bonferroni correction for the HM450K assay, was used to control for multiple testing in any of these EWAS. As other clinical characteristics such as molecular subtype, stage, grade and tumour size were not available in all studies, we did not adjust for them in the EWAS but explored their cross-sectional associations with DNA methylation using the available data, using linear regression models (methylation beta values as the outcome and clinical variables as exposures). This analysis was restricted to the CpGs identified in the primary EWAS (model 2).

The CpGs identified from the primary models and subgroup analyses were assessed in the replication set (165 triple-negatives; 69 events), adjusting for age and stage and stratified by chemotherapy status and cohort site. We also assessed 22 individual methylation markers of survival reported by Kim et al. [9] and De Almeida et al. [8], which we had examined in our previous study [17], using the pooled dataset based on the primary model 1 and five-year survival as the outcome. Replication was defined as having a consistent direction of HR and *P* < 0.05.

#### Pathway analyses

We investigated the biological pathways associated with the two sets of markers identified in model 1 and model 2 of the primary EWAS. This analysis was performed using the Kyoto Encyclopedia of Genes and Genomes (KEGG) database and the *gometh* function from the *missMethyl* package in R [30], controlling for potential bias due to different numbers of CpGs per gene. An FDR q-value < 0.05 was used to identify noteworthy pathways.

#### Machine-learning based signature of survival

To develop and validate a predictive DNA methylation-based signature for five-year overall survival, we used the eight publicly available datasets as the training set (*N* = 1576, N deaths = 225) and the MCCS as the test set (*N* = 425; *N* = 47) as it had more extensive information on clinical characteristics. To avoid potential issues of using too many features in the training sample, we first reduced dimensionality by conducting an EWAS of survival in the training set and retaining CpGs an FDR q-value < 0.05. We applied elastic net-penalised Cox regression (tuning parameter alpha = 0.5) using the *glmnet* package in R, using five-fold cross-validation to select the tuning parameter lambda (one-standard error rule [31]). Elastic net was chosen over least absolute shrinkage and selection operator (LASSO) for its ability to effectively handle high-dimensional data with many correlated features [31, 32], which is relevant to HM450K methylation data. We evaluated the model’s external predictive performance in the MCCS by assessing its association with five-year overall survival with and without adjustment for age, tumour subtype (luminal A, luminal B, HER2-positive, and triple-negative) and tumour stage and calculating the corresponding concordance indices. Among 101 iterations of the elastic net, the model corresponding to the median C-index in the testing set was selected as the final signature and stability across the range of obtained c-indices was examined. Non-parametric bootstrap resampling (10,000 iterations) was used to estimate the difference in c-index between models and its bias-corrected 95% confidence interval (BC) [33].

As a first sensitivity analysis, we trained models with age and ER status incorporated to the penalised Cox model. A second sensitivity analysis included additional adjustment for tumour size and grade and tumour purity (estimated based on DNA methylation data using the *InfiniumPurify* R package [34]), carried out in the MCCS. In the MCCS, subgroup analyses were conducted separately by ER status and tumour stage by fitting Cox models within each group. Effect modification by ER status and tumour stage was assessed using interaction models. Replication of the signature was also examined in the set of triple-negatives (disease-free survival), adjusting for age and stage, and stratified by chemotherapy status and cohort site.

## Results

### Individual methylation markers of survival

The age-adjusted EWAS identified 2,535 CpGs associated with five-year overall survival, Tables S1 and S3a. After adjusting for ER status, this number decreased to 281 CpGs, Table S1 and S3b. These associations were widespread across the genome, Fig. S1. Over 30% of the signals in both models were located in unannotated regions, which was higher than for the whole assay (23%), Table S2.

Of the ten strongest associations in age- and ER status-adjusted models (Table 2), five were annotated to a gene: *NCOR2* (HR per SD = 0.75, 95% confidence interval (CI): 0.69–0.81, *P *= 2 × 10^− 12^), *FRY* (HR *=* 0.64, 95%CI: 0.56–0.72, *P* = 2 × 10^− 12^), *ACAP3* (HR *=* 0.75, 95%CI: 0.70–0.82, *P* = 3 × 10^− 12^), *CAPS* (HR *=* 0.72, 95%CI: 0.65–0.79, *P *= 3 × 10^− 11^) and *NTAN1* (HR *=* 0.64, 95%CI: 0.57–0.73, *P* = 3 × 10^− 11^).

**Table 2:** Ten individual methylation markers with strongest association with the survival outcome

Five-year overall survival (*N* = 1992 cases; N deaths = 272)					Age-adjusted^1^		Age and ER status-adjusted^2^						
CpG	Gene	Chr	Position	Region	HR per SD^3^ (95%CI)	*P*	CpG	Gene	Chr	Position	Region	HR per SD (95%CI)	*P*
cg16461335		4	80,413,489	Non-promoter	0.60 (0.54–0.67)	5.4 × 10^− 20^	cg11601197	*NCOR2*	12	124,812,999	Non-promoter	0.75 (0.69–0.81)	2.0 × 10^− 12^
cg04389921		14	26,226,996	Non-promoter	0.67 (0.61–0.73)	2.9 × 10^− 17^	cg02744945	*FRY*	13	32,738,953	Non-promoter	0.64 (0.56–0.72)	2.3 × 10^− 12^
cg21184711	*CADPS2*	7	122,488,330	Non-promoter	0.65 (0.58–0.72)	5.7 × 10^− 17^	cg17712416	*ACAP3*	1	1,238,436	Non-promoter	0.75 (0.70–0.82)	2.9 × 10^− 12^
cg09289459		14	26,235,384	Non-promoter	0.68 (0.62–0.74)	1.4 × 10^− 16^	cg16461335		4	80,413,489	Non-promoter	0.65 (0.58–0.73)	3.0 × 10^− 12^
cg13883981		3	78,162,085	Non-promoter	0.66 (0.60–0.73)	2.1 × 10^− 16^	cg19640779		13	59,786,086	Non-promoter	0.68 (0.61–0.76)	9.4 × 10^− 12^
cg25268863	*EFEMP1*	2	56,116,062	Non-promoter	0.64 (0.57–0.71)	2.2 × 10^− 16^	cg17800124	*CAPS*	19	5,913,854	Promoter	0.72 (0.65–0.79)	2.8 × 10^− 11^
cg07456681	*NTAN1*	16	15,146,876	Non-promoter	0.60 (0.53–0.67)	2.6 × 10^− 16^	cg07456681	*NTAN1*	16	15,146,876	Non-promoter	0.64 (0.57–0.73)	3.5 × 10^− 11^
cg09610644	*BDH1*	3	197,249,274	Non-promoter	0.67 (0.61–0.74)	2.7 × 10^− 16^	cg11113931		15	40,730,338	Non-promoter	0.66 (0.59–0.75)	5.6 × 10^− 11^
cg09866695		1	221,747,446	Non-promoter	0.62 (0.55–0.70)	2.9 × 10^− 16^	cg03015790		9	96,710,875	Non-promoter	1.44 (1.29–1.61)	7.1 × 10^− 11^
cg05976283		8	103,140,765	Non-promoter	0.63 (0.57–0.71)	5.4 × 10^− 16^	cg13883981		3	78,162,085	Non-promoter	0.71 (0.64–0.78)	7.2 × 10^− 11^

HR, hazard ratio; SD, standard deviation; ER, estrogen receptor; Chr: chromosome

1. Model 1: adjusted for age + stratified by study

2. Model 2: Model 1 + ER status

3. ‘HR per SD’ refers to the hazard ratio per one standard deviation increase in methylation level

Of the ten strongest methylation markers that showed an effect modification by ER status (*P*<1 × 10^− 7^), seven were annotated to a gene: *ADAR* (P effect modification=4 × 10^− 9^), *GPR123* (*P*=2 × 10^− 8^), *CUX1* (*P* =3 × 10^− 8^), *FBRSL1* (*P*=3 × 10^− 8^), *NCKAP1* (*P*=5 × 10^− 8^), *DUSP10* (*P*=7 × 10^− 8^), Table 3. None of these appeared in the primary EWAS (age and ER status-adjusted model), Fig. S2.

**Table 3:** Individual methylation markers with effect modification by ER status for five-year overall survival

Five-year overall survival (*N* = 1992 cases; N deaths = 272)								
CpG	Gene	Chr	Position	Region	All cases	ER-negative	ER-positive	*P* (effect modification by ER status)^2^
					HR per SD^3^ (95% CI)^1^	HR per SD^3^ (95% CI)^1^	HR per SD^3^ (95% CI)^1^	
cg03920003	*ADAR*	1	154,582,007	Promoter	0.92 (0.80–1.06)	1.31 (1.10–1.57)	0.58 (0.48–0.72)	3.8 × 10^− 9^
cg13067553		1	147,782,452	Non-promoter	1.08 (0.95–1.22)	0.77 (0.65–0.92)	1.60 (1.33–1.93)	7.1 × 10^− 9^
cg07505629	*GPR123*	10	134,929,753	Non-promoter	0.93 (0.81–1.06)	0.67 (0.56–0.79)	1.38 (1.14–1.66)	2.0 × 10^− 8^
cg15755348	*CUX1*	7	101,768,874	Non-promoter	0.85 (0.75–0.96)	1.10 (0.94–1.28)	0.57 (0.48–0.67)	2.5 × 10^− 8^
cg00688311	*FBRSL1*	12	133,100,714	Non-promoter	0.90 (0.78–1.03)	1.29 (1.07–1.54)	0.60 (0.50–0.73)	3.3 × 10^− 8^
cg20444370	*NCKAP1*	2	183,875,230	Non-promoter	0.83 (0.73–0.94)	1.07 (0.91–1.26)	0.54 (0.45–0.65)	4.8 × 10^− 8^
cg09135754		1	2,863,146	Non-promoter	0.98 (0.86–1.11)	0.72 (0.61–0.85)	1.44 (1.19–1.74)	6.9 × 10^− 8^
cg03764364		10	29,480,551	Non-promoter	0.79 (0.71–0.88)	1.02 (0.88–1.19)	0.56 (0.49–0.69)	7.3 × 10^− 8^
cg07946977	*DUSP10*	1	221,886,639	Promoter	0.87 (0.77–0.98)	1.10 (0.95–1.29)	0.58 (0.49–0.69)	7.4 × 10^− 8^
cg01165817	*PCCA*	13	101,086,489	Non-promoter	0.93 (0.81–1.06)	1.26 (1.06–1.50)	0.61 (0.50–0.75)	7.8 × 10^− 8^
cg01152374		15	69,817,071	Non-promoter	0.93 (0.83–1.04)	1.14 (0.99–1.33)	0.63 (0.53–0.74)	8.3 × 10^− 8^

HR, hazard ratio; SD, standard deviation; CI; confidence interval; ER, estrogen receptor; Chr: chromosome

1. Model 2: adjusted for age + ER status + stratified by study

2. *P*-effect modification was calculated using likelihood ratio test to compare two models: model 2 and model 2 + ER status × methylation

3. ‘HR per SD’ refers to the hazard ratio per one standard deviation increase in methylation level

### Subgroup analysis by ER status

In ER-positive cases (*N* = 1,377), 389 methylation markers were associated with five-year survival in the age-adjusted model, of which 315 were not found in the primary EWAS (all cases, age and ER status-adjusted model). In ER-negative cases (*N* = 615), 20 methylation markers were associated with five-year survival in the age-adjusted model, of which 11 were not found in the primary EWAS, Table 4.

**Table 4:** Individual methylation markers with the strongest associations for five-year overall survival in ER-positive and ER-negative subgroups, not associated in the primary EWAS (all cases, age and ER-status-adjusted model)

ER-positive: *N* cases = 1377, *N* death = 123							ER-negative: *N* cases = 615, *N* death = 149						
CpG	Gene	Chr	Position	Region	HR per SD^3^ (95%CI)	*P* ^1^	CpG	Gene	Chr	Position	Region	HR per SD^3^ (95%CI)	*P* ^1^
cg04436083	*DHX15*	4	24,567,220	Non-promoter	0.67 (0.60–0.74)	3.5 × 10^− 14^	cg01074413	*LOC100288797*	20	2,796,836	Promoter	0.54 (0.44–0.66)	3.7 × 10^− 9^
cg08024409	*ZNF655*	7	99,155,971	Promoter	1.36 (1.25–1.47)	7.7 × 10^− 14^	cg03269020	*MYO16*	13	109,444,752	Non-promoter	0.65 (0.56–0.76)	1.7 × 10^− 8^
cg26065841	*CHAC1*	15	41,244,853	Promoter	0.61 (0.54–0.70)	5.6 × 10^− 13^	cg02628801	*S1PR1*	1	101,701,963	Promoter	1.39 (1.24–1.56)	1.8 × 10^− 8^
cg12735333	*PIAS1*	15	68,350,548	Non-promoter	0.61 (0.53–0.69)	7.8 × 10^− 13^	cg09869811	*NUTF2*	16	67,897,346	Promoter	0.69 (0.61–0.79)	3.0 × 10^− 8^
cg13636404	*ZNF655*	7	99,155,673	Promoter	1.34 (1.24–1.46)	8.4 × 10^− 13^	cg03625953	*SMG6*	17	1,975,218	Non-promoter	0.57 (0.46–0.69)	3.9 × 10^− 8^
cg02129127	*TAF4*	20	60,635,142	Non-promoter	0.69 (0.62–0.76)	2.9 × 10^− 12^	cg15745944		17	39,861,969	Non-promoter	0.68 (0.59–0.78)	5.6 × 10^− 8^
cg24079830	*MAP3K10*	19	40,702,019	Non-promoter	0.64 (0.57–0.73)	3.6 × 10^− 12^	cg04164415	*ANKRD29*	18	21,199,797	Non-promoter	1.47 (1.28–1.69)	6.1 × 10^− 8^
cg23402524	*SPTBN1*	2	54,885,027	Non-promoter	0.67 (0.59–0.75)	5.6 × 10^− 12^	cg05053688	*S1PR1*	1	101,702,744	Promoter	1.37 (1.22-0.154)	7.0 × 10^− 8^
cg15755348	*CUX1*	7	101,768,874	Non-promoter	0.63 (0.55–0.72)	6.3 × 10^− 12^	cg23782866	*FAM155A*	13	107,928,782	Non-promoter	0.64 (0.54–0.75)	7.2 × 10^− 8^
cg07484673	*ZNF655*	7	99,155,878	Promoter	1.30 (1.21–1.40)	1.0 × 10^− 11^	cg17826986	*DENND4B*	1	153,919,374	Promoter	1.46 (1.27–1.68)	8.0 × 10^− 8^

HR, hazard ratio; SD, standard deviation; ER, estrogen receptor, Chr: chromosome

1. Cox proportional hazard model adjusted for age + stratified by study

### Replication of individual methylation markers of survival

Of the 2,535 associations identified in the primary EWAS (model 1), 1925 (76%) were in the same direction in GSE141441, of which 389 were replicated (*P* < 0.05), Table S3a. Of the 281 markers from the age- and ER status-adjusted EWAS, 221 (79%) were in the same direction and 50 were replicated (*P* < 0.05), Table S3b. Of the 20 associations identified from the EWAS in ER-negative cases, 14 (70%) were in the same direction in the set of triple-negatives, including 3 with *P* < 0.05, Table S3c. Of the 389 associations identified from the EWAS in ER-positive cases, 300 (77%) were in the same direction in the set of triple-negatives, including 82 with *P* < 0.05, Table S3d. Of the 22 previously reported methylation markers by Kim et al. [9] and De Almeida et al. [8], 8 were replicated in the pooled dataset, Table S4.

### Pathway analyses

No KEGG pathways were found to be enriched for either list of CpGs identified from age-adjusted (*N* = 2,535 CpGs) or age- and ER status-adjusted analyses (*N* = 281 CpGs), with the smallest P-value observed for “Biosynthesis of amino acids” (*P* = 0.005, FDR q = 0.97; Table S5).

### Cross-sectional associations with clinical characteristics

For most of the individual DNA methylation markers identified in the primary EWAS (model 2), their association with survival was in the same direction as with tumour size (94%), tumour grade (90%) and stage (76%), Table S6. A high percentage of directionally consistent associations were also observed for ER-negative vs. ER-positive (79%), but not for age (59%) and TNBC vs. luminal A (47%), Table S6.

### Methylation-based signature of survival

The elastic net model selected 228 CpGs, the weights of which are provided in Table S7. This signature was strongly associated with five-year overall survival in the MCCS: (HR per SD = 1.7, 95% CI: 1.2–2.3, *P* = 9.6 × 10^− 4^) after adjustment for age, stage and tumour subtype, with a C-index in this model of 0.79 compared with C-index = 0.75 for the model with clinical variables only, Table 5. This was a C-index increase of 0.04 (95% BC CI: 0.01–0.10, *P* = 0.04). The model with the 228-CpG signature alone (without clinical variables) had a C-index of 0.70, Table S8. There was also evidence of replication in the set of triple-negatives with disease-free survival (GSE141441), with HR per SD = 1.4, 95% CI: 1.1–1.8, *P* = 0.01, after controlling for age, stage, chemotherapy status and cohort site. The C-index was 0.65, compared with 0.57 for the model without the signature, Table 5.

**Table 5:** Accuracy of methylation-based signature of five-year overall survival using all MCCS cases and 165 triple-negative cases from GSE141441

Training set-seven datasets: *N* cases = 1567, *N* death = 225				Test set-MCCS: *N* all cases = 425, *N* death = 47						
N input CpGs	Adjustment	N CpG selected by EWAS^1^	N Methylation signature^2^	Clinical characteristics	Methylation signature + age			Methylation signature + clinical characteristics^3^		
				C-index^3^	HR per SD (95%CI)	*P*	C-index	HR per SD (95%CI)	*P*	C-index
334,578	No	19,070	228	0.75	1.8 (1.4–2.3)	2.6 × 10^− 6^	0.71	1.7 (1.2–2.3)	0.001	0.79
				Test set- GSE141441: N Triple negative cases = 165, N relapse = 69						
				C-index^4^	HR per SD (95%CI)	*P*	C-index	HR per SD (95%CI)	*P* ^4^	C-index
				0.57	1.4 (1.1–1.8)	0.003	0.60	1.4 (1.1–1.8)	0.01	0.65

HR, hazard ratio; CI, confidence interval, SD, standard deviation; ER, estrogen receptor; Training set: TCGA + 7 GEO datasets; C-index: concordance index

1. Number of CpGs identified using EWAS of five-year overall survival: age + ER status + stratified by study on training set based on FDR q-value < 0.05

2. Number of CpGs selected by elastic net regularized Cox proportional hazards regression and five-fold cross-validation method

3. Model adjusted for clinical characteristics using test set: age + stage + subtype (luminal A, luminal B, HER2-positive, triple-negative) + tumour purity

4. Model adjusted for clinical characteristics using test set: age + stage + stratified for cohort site and chemotherapy

In sensitivity analyses, the results were virtually identical in models additionally adjusted for tumour purity, grade and tumour size, Table S8, and when the model was trained on the methylation data with the addition of age and ER status, Table S8. The signature was positively correlated, albeit weakly, with age (*r* = 0.15), tumour size (*r* = 0.19) and tumour purity (*r* = 0.15), Fig. S3. There was no evidence of effect modification by tumour stage (*P* = 0.83) and ER status (*P* = 0.05), the association being greatest for stage II disease (HR per SD = 1.8, 95%CI: 1.2–2.8) and for patients with ER-positive tumours (HR per SD = 1.9, 95%CI: 1.4–2.6), with wide confidence intervals, Table S9.

## Discussion

This study pooled datasets with available genome-wide tumour DNA methylation data to carry out, to our knowledge, the largest EWAS of breast cancer survival to date. Numerous individual methylation markers of five-year overall survival were identified after accounting for age and ER status. A methylation-based signature appeared to provide substantial improvement to survival prediction beyond the main clinical characteristics including age, stage, and immunohistochemistry (IHC)-based subtype. These findings therefore support the potential for DNA methylation to serve as an addition to existing prognostic models of breast cancer survival.

The primary strength of our study was the pooling of data across ten studies with genome-wide DNA methylation and a survival endpoint, including a relatively large number of deaths (*N* = 272 for five-year overall survival). Some analyses with moderate power were previously carried out using TCGA, which includes approximately 100 deaths [8–11, 35]. We carried out the main EWAS using all samples to maximise its ability to detect associations with Bonferroni-significant P-values, but acknowledge that external replication of these signals is required.

For all analyses, replication was investigated in an independent set of triple-negative cases with disease-free survival as the outcome, which represents a biologically distinct subtype and a different survival outcome and is therefore a limitation, although still providing supportive evidence for the robustness of the findings in a high-risk group. Our study used methylation data from multiple cohorts, which may introduce variability that could influence the comparability of results. We minimised this by applying the same processing and normalisation pipeline with raw methylation data available (70% of the cases) but were unable to do so for all studies. This inconsistency, combined with differences in tissue preservation methods (e.g., fresh frozen vs. FFPE), tumour cellularity, and patient demographics, may have contributed to remaining inter-cohort differences. Our analysis was restricted to tumour DNA methylation datasets generated using the Illumina HM450K platform with available survival follow-up. Restricting analyses to a single platform enabled harmonisation of methylation measurements across cohorts to carry out a pooled EWAS. We acknowledge that additional breast cancer methylation datasets exist using other measurement platforms, which were beyond the scope of the present study. Somatic mutations could not be directly accounted for, as tumour-derived genetic data were unavailable for most datasets and the HM450K platform is not designed to detect sequence variation [36]. Consequently, some associations may partly reflect underlying somatic genetic mutations, although epigenetic changes are thought to occur early in carcinogenesis and may precede or contribute to subsequent genomic instability [37, 38]; the impact of underlying somatic mutations should be explored in future studies integrating genomic and epigenomic data. Survival-associated CpGs were distributed across a broad range of genomic contexts, although not enriched in well-annotated regulatory regions, highlighting challenges in functional interpretation using current gene-centric annotation frameworks; further functional characterisation of these signals is required in future studies. The relatively weak pathway enrichment observed may reflect the heterogeneous nature of breast tumours and the widespread distribution of survival-associated methylation changes across multiple biological processes, rather than strong enrichment within specific pathways. In addition, the primary EWAS was designed to maximise power by including all available tumours and conducting more extensively adjusted or stratified analyses in smaller subsets would likely be underpowered for pathway-level inference. Sensitivity analyses incorporating additional clinical variables, including tumour purity, did not materially alter the association between the methylation-based signature and survival, suggesting that confounding due to unavailable clinical variables is unlikely to fully explain the observed patterns.

Additionally, missing data for key clinical characteristics across cohorts, such as IHC-based subtypes, grade, and stage across cohorts restricted our ability to adjust for these variables in both the EWASs and the training of the prediction model. Secondary analyses indicated that these clinical characteristics likely capture a substantial fraction of the methylation signal, which should be investigated in future studies with more comprehensive clinical data. Nevertheless, the CpG signature was strongly associated with survival in the MCCS after comprehensive adjustment for clinical variables (age and tumour subtype, stage, grade, size and purity). We also tested for effect modification by ER status, although these had limited power to detect moderate heterogeneity. In subgroup analyses by ER status, we identified additional epigenome-wide significant candidate signals, suggesting the presence of subtype-specific methylation associations that may be diluted in pooled analyses, although power was limited and replication is also required. Our pooled analysis assumed consistent associations with survival across tumour subtypes, age groups, and cohorts, which may not fully hold. Future studies should formally assess such heterogeneity using well-powered analyses. Larger integrated datasets with standardised protocols and complete clinical annotation particularly with adequate representation of various subtypes (e.g. HER2 positive, triple negative) and other clinically distinct subgroups (e.g. younger patients) are needed to validate subtype-specific findings and improve generalisability. The limited availability of lifestyle and comorbidity data (e.g. obesity) in publicly available tumour methylation datasets constrained more comprehensive confounding assessment. The findings should therefore be interpreted as demonstrating prognostic potential rather than immediate clinical applicability, which would require further testing in large datasets including a greater number of deaths across varied settings.

Of the 22 CpGs previously reported by De Almeida et al. [8] and Kim et al. [9] as associated with breast cancer survival, nine had shown evidence of replication in the MCCS cohort in their original analyses [17]. We re-tested 18 of these in the current study: 8 showed clear evidence of replication and most associations were in the expected direction. These results therefore corroborate our previous findings while also highlighting some heterogeneity of findings across cohorts. Part of the discrepancy between cohorts may lie in differences in the outcome definitions and model adjustments. The original studies used breast cancer-specific and overall survival as the endpoints and compared hazard ratios without adjustment or with adjustment for age, subtype and stage, whereas our EWAS focused on five-year overall survival. Our findings underscore the need for harmonised replication including consistent outcome definitions and variable adjustment to enable meaningful comparison of associations across studies. It also highlights that multi-CpG signatures provide more robust markers of survival than individual CpGs. Among the three previously reported signatures [11–13], two were replicated in the MCCS [17], with the 28-CpG Liu signature showing the strongest association with breast cancer-specific survival in adjusted models [17]. Interestingly, no CpGs were shared between the two signatures, likely reflecting differences in study populations, feature selection procedures, and endpoint definition. Our 228-CpG signature, when added to clinical characteristics, achieved a C-index of 0.79 in the MCCS dataset, reflecting stronger performance than the 28-CpG Liu signature. The higher C-index achieved by our model may be attributable in part to the selection of a greater number of CpGs, possibly due to the larger sample size used for training. The results were very stable across elastic net iterations in terms of observed C-index in the MCCS. Further improvements might be achieved by considering non-linear associations or other machine learning methods. There was weak evidence that the association of the 228-CpG signature with survival differed by ER status, being weaker in ER-negative cases in the MCCS (HR = 1.1). This points to the possibility that the signature may be more prognostically informative in the ER-positive subgroup. Further validation is needed to confirm these observations. The replication of findings in a different context (triple-negative cases and disease-free survival) suggests that while the signature was derived from pooling all subtypes and showed stronger associations in ER-positive disease, some components may have broad prognostic relevance beyond ER status and the specific survival outcome studied. Further validation and potential improvement may still be needed to confirm its clinical utility.

The strongest associations in our primary EWAS included several genes previously linked to breast cancer progression [39–42]. For example, *NCOR2*, a nuclear receptor co-repressor, has been associated with poor prognosis in ER-positive breast cancer and may mediate tamoxifen resistance through transcriptional repression [39]. *FRY*, identified as a potential tumour suppressor, has been shown to inhibit cell proliferation and metastasis in breast cancer cell lines [43]. *ACAP3* has been implicated in breast cancer risk through large-scale transcriptome-wide association studies, with its expression regulated by distal genetic variants (trans-eQTLs) [41]. *CAPS* has been proposed as a predictive marker for endocrine therapy response, with higher protein levels linked to poorer outcomes in ER-positive tumours [31]. These findings suggest that methylation at these loci reflect relevant pathways liking epigenetic processes to tumour progression. There was however no indication that promoter regions were overrepresented and no specific pathways were identified in our pathway enrichment analyses. Finally, our findings also reinforce the notion that subtype-specific biology may influence associations with prognosis [44, 45].

## Conclusion

Using a large dataset of breast tumours, our study found that DNA methylation at many CpGs was associated with five-year overall survival. A 228-CpG epigenetic signature provided improvement in survival prediction beyond the main clinical characteristics, suggesting a role for methylation markers in the prognostication of breast cancer.

## Supplementary Information

Below is the link to the electronic supplementary material.

Supplementary Material 1.

Supplementary Material 2.

## Data Availability

The MCCS individual participant data that support the findings of this study are not publicly available due to privacy and ethical restrictions. The data can be requested by contacting the PEDIGREE resource at [pedigree@cancervic.org.au] . The TCGA-BRCA data were generated by the TCGA Research Network and are publicly available at [https://www.cancer.gov/tcga] . The GEO data sets used in this study are publicly available at the NCBI Gene Expression Omnibus ([https://www.ncbi.nlm.nih.gov/geo/]).

## References

[1] Dworkin AM, Huang TH, Toland AE. Epigenetic alterations in the breast: implications for breast cancer detection, prognosis and treatment. Semin Cancer Biol. 2009;19(3):165–71. 19429480 10.1016/j.semcancer.2009.02.007 PMC2734184

[2] Nishiyama A, Nakanishi M. Navigating the DNA methylation landscape of cancer. Trends Genet. 2021;37(11):1012–27. 34120771 10.1016/j.tig.2021.05.002

[3] Baylin SB, Jones PA. Epigenetic determinants of cancer. Cold Spring Harb Perspect Biol. 2016;8(9):a019505. 27194046 10.1101/cshperspect.a019505 PMC5008069

[4] Feinberg AP, Koldobskiy MA, Göndör A. Epigenetic modulators, modifiers and mediators in cancer aetiology and progression. Nat Rev Genet. 2016;17(5):284–99. 26972587 10.1038/nrg.2016.13 PMC4888057

[5] Feinberg AP, Ohlsson R, Henikoff S. The epigenetic progenitor origin of human cancer. Nat Rev Genet. 2006;7(1):21–33. 16369569 10.1038/nrg1748

[6] Vietri MT, D’elia G, Benincasa G, Ferraro G, Caliendo G, Nicoletti GF, Napoli C. DNA methylation and breast cancer: a way forward. Int J Oncol. 2021;59(5):1–12. 10.3892/ijo.2021.5278 34726251

[7] Davalos V, Martinez-Cardus A, Esteller M. The epigenomic revolution in breast cancer: from single-gene to genome-wide next-generation approaches. Am J Pathol. 2017;187(10):2163–74. 28734945 10.1016/j.ajpath.2017.07.002

[8] de Almeida BP, Apolónio JD, Binnie A, Castelo-Branco P. Roadmap of DNA methylation in breast cancer identifies novel prognostic biomarkers. BMC Cancer. 2019;19(1):219. 30866861 10.1186/s12885-019-5403-0 PMC6416975

[9] Kim DH, Binder AM, Zhou H, Jung SY. DNA methylation patterns associated with breast cancer prognosis that are specific to tumor subtype and menopausal status. Front Genet. 2023;14:1133443. 36936429 10.3389/fgene.2023.1133443 PMC10018014

[10] Peng Y, Shui L, Xie J, Liu S. Development and validation of a novel 15-CpG-based signature for predicting prognosis in triple-negative breast cancer. J Cell Mol Med. 2020;24(16):9378–87. 32649035 10.1111/jcmm.15588 PMC7417707

[11] Tao C, Luo R, Song J, Zhang W, Ran L. A seven-DNA methylation signature as a novel prognostic biomarker in breast cancer. J Cell Biochem. 2020;121(3):2385–93. 31646666 10.1002/jcb.29461

[12] Du T, Liu B, Wang Z, Wan X, Wu Y. CpG methylation signature predicts prognosis in breast cancer. Breast Cancer Res Treat. 2019;178(3):565–72. 31520283 10.1007/s10549-019-05417-3

[13] Liu XP, Hou J, Chen C, Guan L, Hu HK, Li S. A DNA methylation-based panel for the prognosis and dagnosis of patients with breast cancer and its mechanisms. Front Mol Biosci. 2020;7:118. 32733914 10.3389/fmolb.2020.00118 PMC7358612

[14] Pedersen CA, Cao MD, Fleischer T, Rye MB, Knappskog S, Eikesdal HP, Lønning PE, Tost J, Kristensen VN, Tessem M-B, et al. DNA methylation changes in response to neoadjuvant chemotherapy are associated with breast cancer survival. Breast Cancer Res. 2022;24(1):43. 35751095 10.1186/s13058-022-01537-9 PMC9233373

[15] Gao Y, Wang X, Li S, Zhang Z, Li X, Lin F. Identification of a DNA methylation-based prognostic signature for patients with triple-negative breast cancer. Med Sci Monit. 2021;27:e930025. 34003815 10.12659/MSM.930025 PMC8140526

[16] Piazza R, Ramazzotti D, Spinelli R, Pirola A, De Sano L, Ferrari P, Magistroni V, Cordani N, Sharma N, Gambacorti-Passerini C. OncoScore: a novel, Internet-based tool to assess the oncogenic potential of genes. Sci Rep. 2017;7:46290. 28387367 10.1038/srep46290 PMC5384236

[17] Zarean E, Li S, Wong EM, Makalic E, Milne RL, Giles GG, McLean C, Southey MC. Dugué P-A: Tumour DNA methylation markers associated with breast cancer survival: a replication study. Breast Cancer Res. 2025;27(1):9. 39825380 10.1186/s13058-024-01955-x PMC11740461

[18] Hao X, Luo H, Krawczyk M, Wei W, Wang W, Wang J, Flagg K, Hou J, Zhang H, Yi S. DNA methylation markers for diagnosis and prognosis of common cancers. Proc Natl Acad Sci. 2017;114(28):7414–9. 28652331 10.1073/pnas.1703577114 PMC5514741

[19] Xiao B, Chen L, Ke Y, Hang J, Cao L, Zhang R, Zhang W, Liao Y, Gao Y, Chen J. Identification of methylation sites and signature genes with prognostic value for luminal breast cancer. BMC Cancer. 2018;18:1–13. 29642861 10.1186/s12885-018-4314-9 PMC5896050

[20] Zarean E, Li S, Wong EM, Makalic E, Milne RL, Giles GG, McLean C, Southey MC. Dugué P-A: Evaluation of agreement between common clustering strategies for DNA methylation-based subtyping of breast tumours. Epigenomics. 2025;17(2):105–14. 39711216 10.1080/17501911.2024.2441653 PMC11792870

[21] Network CGA. Comprehensive molecular portraits of human breast tumours. Nature. 2012;490(7418):61. 23000897 10.1038/nature11412 PMC3465532

[22] Xu Z, Niu L, Li L, Taylor JA. ENmix: a novel background correction method for Illumina HumanMethylation450 BeadChip. Nucleic Acids Res. 2016;44(3):e20–20. 26384415 10.1093/nar/gkv907 PMC4756845

[23] Xu Z, Niu L, Taylor JA. The ENmix DNA methylation analysis pipeline for Illumina BeadChip and comparisons with seven other preprocessing pipelines. Clin epigenetics. 2021;13(1):216. 34886879 10.1186/s13148-021-01207-1 PMC8662917

[24] Ori AP, Lu AT, Horvath S, Ophoff RA. Significant variation in the performance of DNA methylation predictors across data preprocessing and normalization strategies. Genome Biol. 2022;23(1):225. 36280888 10.1186/s13059-022-02793-w PMC9590227

[25] Chen YA, Lemire M, Choufani S, Butcher DT, Grafodatskaya D, Zanke BW, Gallinger S, Hudson TJ, Weksberg R. Discovery of cross-reactive probes and polymorphic CpGs in the Illumina Infinium HumanMethylation450 microarray. Epigenetics. 2013;8(2):203–9. 23314698 10.4161/epi.23470 PMC3592906

[26] Benton MC, Johnstone A, Eccles D, Harmon B, Hayes MT, Lea RA, Griffiths L, Hoffman EP, Stubbs RS, Macartney-Coxson D. An analysis of DNA methylation in human adipose tissue reveals differential modification of obesity genes before and after gastric bypass and weight loss. Genome Biol. 2015;16:1–21. 25651499 10.1186/s13059-014-0569-x PMC4301800

[27] Zhou W, Laird PW, Shen H. Comprehensive characterization, annotation and innovative use of Infinium DNA methylation BeadChip probes. Nucleic Acids Res. 2017;45(4):e22. 27924034 10.1093/nar/gkw967 PMC5389466

[28] Hastie T, Tibshirani R, Sherlock G, Eisen M, Brown P, Botstein D. Imputing missing data for gene expression arrays. 1999.

[29] Bellera CA, MacGrogan G, Debled M, de Lara CT, Brouste V, Mathoulin-Pélissier S. Variables with time-varying effects and the Cox model: some statistical concepts illustrated with a prognostic factor study in breast cancer. BMC Med Res Methodol. 2010;10(1):20. 20233435 10.1186/1471-2288-10-20 PMC2846954

[30] Phipson B, Maksimovic J, Oshlack A. missMethyl: an R package for analyzing data from Illumina’s HumanMethylation450 platform. Bioinformatics. 2016;32(2):286–8. 26424855 10.1093/bioinformatics/btv560

[31] Breiman L, Friedman J, Olshen RA, Stone CJ. Classification and regression trees: Routledge; 2017.

[32] Zou H, Hastie T. Regularization and variable selection via the elastic net. J Royal Stat Soc Ser B: Stat Methodol. 2005;67(2):301–20.

[33] Efron B, Narasimhan B. The automatic construction of bootstrap confidence intervals. J Comput Graphical Stat. 2020;29(3):608–19. 10.1080/10618600.2020.1714633 PMC7958418 33727780

[34] Qin Y, Feng H, Chen M, Wu H, Zheng X. InfiniumPurify: An R package for estimating and accounting for tumor purity in cancer methylation research. Genes Dis. 2018;5(1):43–5. 30258934 10.1016/j.gendis.2018.02.003 PMC6147081

[35] Liu X-P, Hou J, Chen C, Guan L, Hu H-K, Li S. A DNA methylation-based panel for the prognosis and diagnosis of patients with breast cancer and its mechanisms. Front Mol Biosci. 2020;7:118. 32733914 10.3389/fmolb.2020.00118 PMC7358612

[36] Jia P, Zhao Z. Impacts of somatic mutations on gene expression: an association perspective. Brief Bioinform. 2016;18(3):413–25. 10.1093/bib/bbw037 PMC5862283 27127206

[37] Sawan C, Vaissière T, Murr R, Herceg Z. Epigenetic drivers and genetic passengers on the road to cancer. Mutat Res - Fundamental Mol Mech Mutagen. 2008;642(1):1–13. 10.1016/j.mrfmmm.2008.03.002 18471836

[38] Sharma S, Kelly TK, Jones PA. Epigenetics in cancer. Carcinogenesis. 2009;31(1):27–36. 19752007 10.1093/carcin/bgp220 PMC2802667

[39] Tsai KK, Huang SS, Northey JJ, Liao WY, Hsu CC, Cheng LH, Werner ME, Chuu CP, Chatterjee C, Lakins JN, et al. Screening of organoids derived from patients with breast cancer implicates the repressor NCOR2 in cytotoxic stress response and antitumor immunity. Nat Cancer. 2022;3(6):734–52. 35618935 10.1038/s43018-022-00375-0 PMC9246917

[40] Liu Y, Chen X, Gong Z, Zhang H, Fei F, Tang X, Wang J, Xu P, Zarbl H, Ren X. Fry is required for mammary gland development during pregnant periods and affects the morphology and growth of breast cancer cells. Front Oncol. 2019;9:1279. 31824855 10.3389/fonc.2019.01279 PMC6881260

[41] Head ST, Dezem F, Todor A, Yang J, Plummer J, Gayther S, Kar S, Schildkraut J, Epstein MP. Cis- and trans-eQTL TWASs of breast and ovarian cancer identify more than 100 susceptibility genes in the BCAC and OCAC consortia. Am J Hum Genet. 2024;111(6):1084–99. 38723630 10.1016/j.ajhg.2024.04.012 PMC11179407

[42] Johansson HJ, Sanchez BC, Forshed J, Stål O, Fohlin H, Lewensohn R, Hall P, Bergh J, Lehtiö J, Linderholm BK. Proteomics profiling identify CAPS as a potential predictive marker of tamoxifen resistance in estrogen receptor positive breast cancer. Clin Proteomics. 2015;12(1):8. 25878567 10.1186/s12014-015-9080-y PMC4389343

[43] Kim J, Kim S, Ko S, In YH, Moon HG, Ahn SK, Kim MK, Lee M, Hwang JH, Ju YS, et al. Recurrent fusion transcripts detected by whole-transcriptome sequencing of 120 primary breast cancer samples. Genes Chromosomes Cancer. 2015;54(11):681–91. 26227178 10.1002/gcc.22279

[44] de Ruijter TC, van der Heide F, Smits KM, Aarts MJ, van Engeland M, Heijnen VCG. Prognostic DNA methylation markers for hormone receptor breast cancer: a systematic review. Breast Cancer Res. 2020;22(1):13. 32005275 10.1186/s13058-020-1250-9 PMC6993426

[45] Guo T, Ren Y, Wang B, Huang Y, Jia S, Tang W, Luo Y. Promoter methylation of BRCA1 is associated with estrogen, progesterone and human epidermal growth factor receptor–negative tumors and the prognosis of breast cancer: A meta-analysis. Mol Clin Oncol. 2015;3(6):1353–60. 26807247 10.3892/mco.2015.620 PMC4665309

