# Supplementary material for: Tumour-based DNA methylation markers of breast cancer survival: a pooled analysis of 2157 cases
Source: Breast Cancer Res. 2026 Jun 26;28:155. doi: 10.1186/s13058-026-02327-3 (PMC13579885; doi:10.1186/s13058-026-02327-3)
Supplement: Supplementary file 1 [file 13058_2026_2327_MOESM2_ESM.docx]

**Supplementary materials:** Tumour-based DNA methylation markers of breast cancer survival: A pooled analysis of 2,157 cases

**Methods**

**Supplementary tables**

**Table S1**. Number of CpG associated with the survival outcome

**Table S2.** The distribution of methylation makers within promoter regions.

**Table S3a**. Replication of individual methylation markers of survival identified in primary EWAS age-adjusted model in GSE141441 dataset.

**Table S3b.** Replication of individual methylation markers of survival identified in primary EWAS age and ER status-adjusted model in GSE141441 dataset.

**Table S3c.** Replication of individual methylation markers of survival identified in subgroup analysis of ER-negative cases in GSE141441 dataset.

**Table S3d.** Replication of individual methylation markers of survival identified in subgroup analysis of ER-positive cases in GSE141441 dataset.

**Table S4.** Evaluating the replicability of previously reported individual methylation markers of survival in the primary EWAS (model1).

**Table S5.** Top five pathways for strongest methylation markers associated with five-year overall survival using KEGG analysis.

**Table S6**. Cross-sectional associations between individual DNA methylation markers and clinical characteristics for those strongly associated with survival.

**Table S**7. 228-CpG signature of survival after adjusted for age, stage, and tumour subtypes.

**Table S8.** Prediction performance of DNA methylation-based signature after adjusting for clinical characteristics.

**Table S9.** Association between DNA methylation-based signature and 5-year survival by ER status and tumour stage in the MCCS.

**Supplementary Figures**

**Figure S1.** Individual methylation markers of five-year overall survival identified by two adjusted models.

**Figure S2.** Individual methylation markers of five-year survival and effect modification by ER-positivity status.

**Figure S3.** Distribution of the 228-CpG survival signature by clinical characteristics.

**Methods**

Ten independent datasets that included DNA methylation measures in breast tumour samples and survival outcome data were pooled. The Melbourne Collaborative Cohort Study (MCCS) was used as the primary dataset and complemented by eight publicly available datasets: The Cancer Genome Atlas (TCGA) and seven datasets available from the Gene Expression Omnibus (GEO) (GSE72245, GSE72251, GSE72254, GSE37754, GSE69914, GSE78754, GSE75067, and GSE141441. All datasets used the Illumina HumanMethylation450 (HM450) BeadChip assay for DNA methylation measurement. We provide below a summary of the cohort characteristics, quality control steps and normalization methods.

*Melbourne Collaborative Cohort Study (MCCS)*

The Melbourne Collaborative Cohort Study (MCCS) recruited 41,513 participants of European ancestry, aged 40-69, in 1990-1994 to study the impact of dietary and lifestyle factors on cancer progression and chronic diseases [1]. Formalin-fixed paraffin-embedded (FFPE) tumour samples from participants diagnosed with invasive breast cancer were analysed using the Illumina Infinium HumanMethylation450K (HM450) BeadChip array. Quality control procedures ensured that 90% of the samples met criteria for DNA integrity and bisulfite conversion [2, 3]. Methylation data were pre-processed and normalized using the *ENmix* pipeline, which performs background correction, dye-bias adjustment, and probe-type bias correction [4]. Probes and samples were removed if the detection P-value exceeded 0.01 in more than 5% of cases. After excluding five samples with incomplete stage or IHC-based molecular subtype data, the final dataset included 425 samples and 476,155 CpG sites.

*The Cancer Genome Atlas (TCGA)*

DNA methylation data (HM450) for breast cancer were obtained from the TCGA portal (https://portal.gdc.cancer.gov/). The dataset included clinical information and methylation profiles for 797 breast tumour samples and 97 normal samples [5]. We excluded normal samples, male cases (N=7), metastatic cases (N=5), and cases without available methylation data (N=52). After these exclusions, the final dataset comprised 731 tumour samples. The raw methylation data were normalized using the same approach applied to the MCCS dataset, resulting in a dataset with 473,646 probes.

*GSE72308*

The GSE72308 dataset includes three cohorts: GSE72245, GSE72251, and GSE72254, providing methylation data (HM450) for 295 breast tumours, including 52 patients with all-cause mortality. Cohort 1 (GSE72245) includes 118 tumour samples from patients diagnosed between 1995 and 2003, while Cohort 2 (GSE72251) contains 119 tumour samples from patients diagnosed between 2004 and 2009, consisting of retrospectively selected fresh-frozen tumours from patients treated with adjuvant therapies. The third cohort, GSE72254, known as the Trial of Principle (TOP) cohort, includes 58 ER-negative patients treated at the same institute between 2003 and 2008, all of whom received neoadjuvant epirubicin monotherapy before surgery [6]. Methylation data from these GEO datasets were normalized using the same approach as used in the MCCS.

*GSE37754*

This dataset includes DNA methylation data (HM450) from 62 fresh-frozen breast tumour samples, previously analysed in multiple studies [7-9]. The samples were collected from African-American and European-American patients residing in Baltimore, Maryland, recruited between 1993 and 2003, as previously described. Patients were identified through surgery lists at hospitals including the University of Maryland Medical Centre, the Baltimore Veterans Affairs Medical Centre, Union Memorial Hospital, Mercy Medical Centre, and Sinai Hospital and were enrolled into the study prior to surgery [10]. The raw methylation data were processed and normalized using the *lumi* package in R, as described in the original study [7].

*GSE69914*

This methylation dataset (HM450) originates from a German cohort study, the Bavarian Breast Cancer Cases and Controls Study 2. It includes data from 308 breast tumour samples, with 254 classified as estrogen receptor (ER)-positive and 48 as ER-negative. The dataset, consisting of 485,512 CpG probes, has been used in multiple studies (e.g., Teschendorff et al. [11, 12], Gao et al. [13, 14], Yang et al. [15]). Raw data were normalized using the *minfi* Bioconductor package as described by Teschendorff et al. Probes with detection P-values larger than 0.05 were assigned to missing and subsequently imputed using the KNN algorithm, resulting in a dataset of 485,512 probes and 308 samples [11].

*GSE78754*

This dataset included DNA methylation data (HM450) from 70 invasive ductal carcinomas triple-negative breast cancers (TNBCs) patients of which 31 patients experienced death due to breast cancer [16], from the Australian Breast Cancer Tissue Bank [17]. Raw data were normalized using Illumina normalization in Genomic Suite 6.6 (Partek) [16], which resulted in a matrix of 485,512 probes and 70 samples.

*GSE75067*

This dataset included 188 breast tumours collected from the Southern Sweden Breast Cancer Group tissue bank at the Department of Oncology and Pathology, Skåne University Hospital (Lund, Sweden), and from the Department of Pathology, Landspitali University Hospital (Reykjavik, Iceland) [18]. DNA methylation data (HM450) were normalized using peak-based correction with Epanechnikov kernel smoothing to adjust for Infinium I and II probe biases, aligning unmethylated and methylated peaks to 0 and 1, respectively [18, 19].

*GSE141441*

This dataset included FFPE tumour samples from 279 triple-negative breast cancer (TNBC) cases collected from Johns Hopkins University, Duke University Medical Center, the University of Iowa, and the International Breast Cancer Study Group (IBCSG) [20]. Quality control steps included exclusion of samples with DNA quantity below 300 ng, high detection p-values (P > 0.01), and those with a percentage of probes passing QC that was more than 2.5 standard deviations below the average across all samples and imported in the Partek Genomics Suite [20].

|  | | EWAS of survival | | Effect modification |
| --- | --- | --- | --- | --- |
|  |  | Adjusted for age^1^ | Adjusted for age and ER status^2^ | ER status^3^ |
| Five-year all-cause deaths (N = 272) | Bonferroni significant | 2,535 | 281 | 11 |
|  | FDR q-value <0.05 | 47,954 | 24,912 | 452 |

**Table S1.** Number of CpG associated with the survival outcome.

ER, estrogen receptor; EWAS, Epigenome-wide association study

Bonferroni-adjusted significance threshold (P < 1x10^-7^) and FDR-q-value<0.05 were used to identify individual methylation markers.

1. Model 1: Adjusted for age + stratified by study
2. Model 2: Model 1+ ER status
3. Model 2 + methylation marker × ER status

| Probes | Promoter region, N (%) | | | | Non-promoter regions, N (%) | | | Total |
| --- | --- | --- | --- | --- | --- | --- | --- | --- |
|  | TSS200 | TSS1500 | 5'UTR | 1stExon | Body | 3'UTR | Not annotated |  |
| All CpGs | 35858 (10.7%) | 49355 (14.8) | 30623 (9.2) | 16093 (4.8) | 114034 (34.1) | 12416 (3.7) | 76199 (22.8) | 334578 |
| Promoter associated probes | 18319 (28.3%) | 16332 (25.2) | 8921 (13.8) | 6348 (9.8) | 10426 (16.1) | 500 (0.8) | 3851 (6.0) | 64697 |
| Signals; age-adjusted model^1^ | 126 (5.0%) | 322 (12.7) | 201 (7.9) | 63 (2.5) | 957 (37.8) | 93 (3.7) | 773 (30.5) | 2535 |
| Signals; age and ER status-adjusted model^2^ | 11 (3.9%) | 35 (12.5) | 21 (7.5) | 13 (4.6) | 104 (37.0) | 12 (4.3) | 85 (30.2) | 281 |

**Table S2.** The distribution of methylation makers within promoter regions**.**

ER, estrogen receptor

1. Model 1: Adjusted for age + stratified by study
2. Model 2: Model 1+ ER status

The following supplementary tables are attached as excel files

**Table S3a.** Replication of individual methylation markers of survival identified in primary EWAS age-adjusted model in GSE141441 dataset.

**Table S3b.** Replication of individual methylation markers of survival identified in primary EWAS age and ER status-adjusted model in GSE141441 dataset.

**Table S3c.** Replication of individual methylation markers of survival identified in subgroup analysis of ER-negative cases in GSE141441 dataset.

**Table S3d.** Replication of individual methylation markers of survival identified in subgroup analysis of ER-positive cases in GSE141441 dataset.

**Table S4.** Replication of previously reported individual methylation markers of survival in the primary EWAS (model1).

|  | | | | MCCS | | Pooled dataset | |
| --- | --- | --- | --- | --- | --- | --- | --- |
| CpG | Study | Gene | Direction^1^ | HR (95% CI) | P^2^ | HR (95% CI) | P^3^ |
| cg01268824 | De Almeida et al. [21] | *ZNF154* | + | 1.18 (0.93,1.51) | 0.18 | 1.12 (0.99, 1.27) | 0.08 |
| cg22674699 |  | *HOXD9* | + | 1.46 (1.11,1.91) | 0.01 | **1.29 (1.13, 1.47)** | **2.2 x 10^-4^** |
| cg12374721 |  | *C17orf93* | + | 1.58 (1.21,2.07) | 0.001 | NA |  |
| cg18081940 |  | *TDRD10* | + | 1.41 (1.08,1.85) | 0.01 | **1.42 (1.23, 1.63)** | **1.5 x 10^-6^** |
| cg04475027 |  | *TMEM132C* | + | 1.26 (0.97,1.63) | 0.08 | 1.13 (0.99, 1.30) | 0.07 |
| cg03985718 | Kim et al. [22] | *TGFBRAP1* | - | 1.16 (0.90,1.51) | 0.25 | 0.91 (0.76, 1.08) | 0.26 |
| cg04921068 |  | *PPM1L* | - | 0.86 (0.65,1.13) | 0.28 | NA |  |
| cg15462203 |  | *DVL1* | - | 0.92 (0.74,1.15) | 0.46 | 0.86 (0.70, 1.05) | 0.14 |
| cg17827670 |  | *AHCYL2* | - | 0.64 (0.46,0.90) | 0.01 | 1.06 (0.94, 1.20) | 0.32 |
| cg09926728 |  | *SH3PXD2A* | - | 0.86 (0.71,1.03) | 0.11 | 0.94 (0.82, 1.07) | 0.34 |
| cg18703983 |  | *KCNS3* | - | 0.74 (0.62,0.89) | 0.001 | **0.80 (0.72, 0.89)** | **8.1 x 10^-5^** |
| cg16976520 |  | *ESYT2* | - | 0.89 (0.71,1.11) | 0.29 | NA |  |
| cg17735983 |  | *MZF1* | + | 1.34 (1.14,1.59) | 0.001 | **1.23 (1.14, 1.34)** | **2.6 x 10^-7^** |
| cg10678486 |  | *ELAC1* | + | 1.18 (1.01,1.38) | 0.04 | 1.08 (0.98, 1.18) | 0.13 |
| cg13447284 |  |  | - | 1.08 (0.84,1.39) | 0.54 | 0.89 (0.73, 1.10) | 0.29 |
| cg24328142 |  | *TSPAN15* | - | 0.76 (0.62,0.95) | 0.01 | **0.74 (0.66, 0.83)** | **2.2 x 10^-7^** |
| cg03216043 |  | *DNM2* | - | 1.09 (0.84,1.40) | 0.52 | **0.82 (0.73, 0.92)** | **0.008** |
| cg22776912 |  | *TMC3* | - | 1.01 (0.79,1.29) | 0.94 | **0.76 (0.67, 0.85)** | **4.0 x 10^-6^** |
| cg06956006 |  | *ACLY* | - | 0.99 (0.78,1.25) | 0.91 | NA |  |
| cg00175150 |  | *ECM1* | - | 0.84 (0.70,1.01) | 0.07 | 0.84 (0.70, 1.01) | 0.07 |
| cg15348839 |  |  | - | 0.98 (0.78,1.24) | 0.88 | 0.87 (0.74, 1.01) | 0.07 |
| cg12511487 |  |  | - | 0.81 (0.66,1.00) | 0.05 | **0.83 (0.74, 0.93)** | **0.001** |

HR: hazard ratio; CI: confidence interval; NA: not available in the pooled dataset.

1. Direction of coefficients as reported in Kim et al. and De Almeida et al.
2. Model 0: unadjusted [23]
3. Model 1: Adjusted for age + stratified by study

**Table S5.** Top five pathways for strongest methylation markers associated with five-year overall survival using KEGG analysis.

| Model 1^1^ (N=2,535 CpGs) | | | | | |
| --- | --- | --- | --- | --- | --- |
| Pathway | N Pathway size | N DEG | P | FDR | Gene |
| Biosynthesis of amino acids | 71 | 11 | 0.005 | 0.97 | *ENO1*, *ENO2*, *PHGDH*, *PYCR2*, *ASS1*, *PGAM1*, *BCAT1*, *SHMT2*, *TKTL2*, *GPT2* |
| Adipocytokine signalling pathway | 70 | 12 | 0.01 | 0.97 | *AKT2*, *NPY*, *PRKAG2*, *MAPK10*, *RXRA*, *STAT3*, *TNFRSF1B*, *ACSBG2*, *TRADD*, *SOCS3*, *CD36* |
| cAMP signalling pathway | 225 | 29 | 0.01 | 0.97 | *RAPGEF4*, *CHRM1*, *ADORA2A*, *AKT2*, *GHSR*, *GIPR*, *GLI3*, *GNAS*, *GRIN2D*, *HTR1E*, *HTR4*, *KCNK2*, *LIPE*, *NFATC1*, *ATP1B2*, *NPY*, *ATP2B4*, *PAK1*, *PLCE1*, *PDE4C*, *PLD1*, *CNGB3*, *PRKACA*, *MAPK10*, *CREB3L2*, *TIAM1*, *VAV2*, *CACNA1D*, *ACOX3* |
| Fc gamma R-mediated phagocytosis | 96 | 15 | 0.02 | 0.97 | *ARPC1B*, *PLA2G4E*, *AKT2*, *GSN*, *LYN*, *MYO10*, *PAK1*, *PLD1*, *PRKCE*, *PRKCG*, *VAV2*, *PIP5K1A*, *PLPP3*, JMJD*7-PLA2G4B*, *ASAP2* |
| Endocrine and other factor-regulated calcium reabsorption | 53 | 10 | 0.02 | 0.97 | *ESR1*, *DNM3*, *GNAS*, *ATP1B2*, *ATP2B4*, *PRKACA*, *PRKCG*, *SLC8A1*, *VDR* |
| Model 2^2^ (N=281 CpGs) | | | | | |
| Leukocyte transendothelial migration | 112 | 5 | 0.01 | 1 | *CTNNA2*, *ITGA4*, *ITGAM*, *ITGB2*, *VAV2* |
| Hematopoietic cell lineage | 93 | 4 | 0.01 | 1 | *ITGA4*, *ITGAM*, *CD14*, *CD36* |
| Legionellosis | 56 | 3 | 0.02 | 1 | *ITGAM*, *ITGB2*, *CD14* |
| Regulation of actin cytoskeleton | 76 | 3 | 0.02 | 1 | *ITGAM*, *ITGB2*, *CD14* |
| Pertussis | 230 | 7 | 0.02 | 1 | *BAIAP2*, *ITGA4*, *ITGAM*, *ITGB2*, *RDX*, *VAV2*, *DIAPH3* |

FDR, false discovery rate q-value<0.05; DEG, differentially expressed genes; ER, estrogen receptor

1. Pathway enrichment of 795 CpGs associated with survival based on Cox proportional model adjusted for age + stratified for study.
2. Pathway enrichment of 60 CpGs associated with survival based on Cox proportional model adjusted for age + ER status+ stratified for study.

**Table S6.** Cross-sectional associations between individual DNA methylation markers and clinical characteristics for those strongly associated with survival.

| Variable | Same direction (%) | P ^1^ < 1.8x10^-4^ (%) |
| --- | --- | --- |
| Age | 165 (58.7) | 53 (32.1) |
| Stage | 213 (75.8) | 86 (40.4) |
| Grade | 252 (89.7) | 190 (75.4) |
| Tumour size | 264 (94.0) | 129 (48.9) |
| ER negative vs ER positive | 221 (78.6) | 164 (74.2) |
| Triple-negative vs luminal A | 132 (47.0) | 37 (28.0) |
| Luminal B vs luminal A | 31 (11.0) | 0 (0.0) |
| HER2-positive vs luminal A | 68 (24.2) | 0 (0.0) |

Note: Individual DNA methylation markers (N=281) from model 2 (adjusted for age and ER status and stratified by study with P<1x10^-7^) were selected.

1. P-value threshold was defined using the Bonferroni correction for the number of CpGs tested: P < 0.05 / 281.

**Table S7** is attached as an excel file.

**Table S7**. 228-CpG signature of survival after adjusted for age, stage, and tumour subtypes.

| Training set-seven datasets: N cases= 1567, N death= 225 | | | | Test set-MCCS: N all cases=425, N death=47 | | | |
| --- | --- | --- | --- | --- | --- | --- | --- |
| N input CpGs | Adjustment | N CpG selected by EWAS^1^ | N Methylation Signature^2^ | Model | C-index | Methylation signature,  HR per SD (95%CI) | P |
|  |  |  |  | Model 1^a^ | 0.75 |  |  |
|  |  |  |  | Model 2^b^ | 0.75 |  |  |
|  |  |  |  | Model 3^c^ | 0.75 |  |  |
|  |  |  |  | Model 4^d^ | 0.75 |  |  |
| 334,578 | No | 19,070 | 228 | 228-CpG signature alone | 0.70 | 1.9 (1.5-2.4) | 5.2 x 10^-7^ |
|  |  |  | 228 | 228-CpG signature + Model 1 | 0.79 | 1.6 (1.2-2.2) | 0.001 |
|  |  |  | 228 | 228-CpG signature + Model 2 | 0.79 | 1.6 (1.2-2.1) | 0.001 |
|  |  |  | 228 | 228-CpG signature + Model 3 | 0.79 | 1.6 (1.2-2.1) | 0.001 |
|  |  |  | 228 | 228-CpG signature + Model 4 | 0.79 | 1.5 (1.1-2.1) | 0.008 |
|  | Age + ER status |  | 226 | 226-CpG signature alone + age^4^ | 0.72 | 1.8 (1.4-2.4) | 2.3 x 10^-6^ |
|  |  |  | 226 | 226-CpG signature + Model 2 | 0.79 | 1.6 (1.2-2.2) | 0.001 |

**Table S8.** Prediction performance of DNA methylation-based signature after adjusting for clinical characteristics.

HR: hazard ratio; CI: confidence interval; C-index: concordance index, SD: standard deviation; ER: estrogen receptor; Training set: TCGA + 7 GEO datasets;

a) Model 1: adjusted for age + stage + tumour subtype
b) Model 2: Model 1 + tumour purity
c) Model 3: Model 1 + tumour purity + tumour grade
d) Model 4: Model 1 + tumour purity + tumour grade + tumour size
1. Number of CpGs identified using EWAS of five-year overall survival: age + ER status + stratified by study on training set based on FDR q-value <0.05.
2. Number of CpGs selected by elastic net regularized Cox proportional hazards regression and five-fold cross-validation method.
3. Methylation signature (per SD), calculated using methylation values of selected CpGs for 5-year overall survival.
4. Methylation-based signature adjusted for age and stratified by study.

**Table S9.** Association between DNA methylation-based signature and 5-year survival by ER status and tumour stage in the MCCS.

| Variable | Model | Epigenetic signature  HR per SD (95% CI) | P-interaction |
| --- | --- | --- | --- |
| ER status |  |  |  |
| ER negative | 228-CpG signature ^a^ + adjusted model ^b^ | 1.1 (0.5-2.1) | 0.05 |
| ER positive | 228-CpG signature + adjusted model ^b^ | 1.9 (1.4-2.6) |  |
|  |  |  |  |
| Tumour stage |  |  |  |
| Stage I | 228-CpG signature + adjusted model ^c^ | 1.6 (0.9-2.6) | 0.83 |
| Stage II | 228-CpG signature + adjusted model ^c^ | 1.8 (1.2-2.8) |  |
| Stage III/IV | 228-CpG signature + adjusted model ^c^ | 1.6 (0.9-2.6) |  |

HR: hazard ratio; CI: confidence interval; C-index: concordance index; SD: standard deviation; MCCS: Melbourne Collaborative Cohort Study, ER: estrogen status.

a) Methylation signature derived from Table S8
b) Adjusted for age+ tumour stage + tumour purity
c) Adjusted for age + tumour subtype + tumour purity

**Figure S1.** Individual methylation markers of five-year overall survival identified by two adjusted models.

Manhattan plots illustrate the association of individual DNA methylation markers with five-year overall survival based on two models: i) adjusted for age and stratified by study and ii) model 1 + ER status. X- axis displays CpG sites along the chromosome’s locations. Each chromosome is assigned a unique colour, highlighting the genomic context of the associations. Chromosome numbers on the x-axis range from 1 to 22, corresponding to the genomic position of each CpG. Y-axis displays the p-value threshold of their association based on: -log10(p-value). The dashed horizontal line indicates the Bonferroni-adjusted significance threshold (P < 1x10^-7^), marking CpG sites associated with the outcome.

**Figure S2.** Individual methylation markers of five-year survival and effect modification by ER-positivity status.

Volcano plot displays the individual methylation markers of five-year overall survival. Green dots represent survival-associated CpGs identified based on model 2: age+ ER status+ stratified by study. Red dots represent methylation markers with effect modification by ER status identified based on model 2+ methylation markers × ER status and selected based Bonferroni p-value threshold of P for effect modification <1x10^-7^. X-axis shows the log hazard ratios for individual associations of DNA methylation markers with the survival. The dashed horizontal line indicates the Bonferroni-adjusted significance threshold (P<1x10^-7^).

**Figure S3.** Distribution of the 228-CpG survival signature by clinical characteristics.

Figure S3 displays the linear relationship between the signature and tumour purity, age, and tumour size. This relationship was checked using Pearson correlation test. The correlation coefficient (r), 95%CI and the P-value were reported for each test within the plots.

**References**

1.	Milne RL, Fletcher AS, MacInnis RJ, Hodge AM, Hopkins AH, Bassett JK, Bruinsma FJ, Lynch BM, Dugué PA, Jayasekara H *et al*: **Cohort Profile: The Melbourne Collaborative Cohort Study (Health 2020)**. *Int J Epidemiol* 2017, **46**(6):1757-1757i.

2.	Wong EM, Joo JE, McLean CA, Baglietto L, English DR, Severi G, Hopper JL, Milne RL, FitzGerald LM, Giles GG *et al*: **Tools for translational epigenetic studies involving formalin-fixed paraffin-embedded human tissue: applying the Infinium HumanMethyation450 Beadchip assay to large population-based studies**. *BMC Res Notes* 2015, **8**:543.

3.	Wong EM, Joo JE, McLean CA, Baglietto L, English DR, Severi G, Wu HC, Terry MB, Hopper JL, Milne RL *et al*: **Analysis of the breast cancer methylome using formalin-fixed paraffin-embedded tumour**. *Breast Cancer Res Treat* 2016, **160**(1):173-180.

4.	Xu Z, Niu L, Taylor JA: **The ENmix DNA methylation analysis pipeline for Illumina BeadChip and comparisons with seven other preprocessing pipelines**. *Clinical epigenetics* 2021, **13**(1):216.

5.	Network CGA: **Comprehensive molecular portraits of human breast tumours**. *Nature* 2012, **490**(7418):61.

6.	Jeschke J, Bizet M, Desmedt C, Calonne E, Dedeurwaerder S, Garaud S, Koch A, Larsimont D, Salgado R, Van den Eynden G: **DNA methylation–based immune response signature improves patient diagnosis in multiple cancers**. *The Journal of clinical investigation* 2017, **127**(8):3090-3102.

7.	Terunuma A, Putluri N, Mishra P, Mathé EA, Dorsey TH, Yi M, Wallace TA, Issaq HJ, Zhou M, Killian JK: **MYC-driven accumulation of 2-hydroxyglutarate is associated with breast cancer prognosis**. *The Journal of clinical investigation* 2014, **124**(1):398-412.

8.	Putluri N, Maity S, Kommagani R, Creighton CJ, Putluri V, Chen F, Nanda S, Bhowmik SK, Terunuma A, Dorsey T: **Pathway-centric integrative analysis identifies RRM2 as a prognostic marker in breast cancer associated with poor survival and tamoxifen resistance**. *Neoplasia* 2014, **16**(5):390-402.

9.	Tang W, Zhou M, Dorsey TH, Prieto DA, Wang XW, Ruppin E, Veenstra TD, Ambs S: **Integrated proteotranscriptomics of breast cancer reveals globally increased protein-mRNA concordance associated with subtypes and survival**. *Genome medicine* 2018, **10**:1-14.

10.	Boersma BJ, Howe TM, Goodman JE, Yfantis HG, Lee DH, Chanock SJ, Ambs S: **Association of Breast Cancer Outcome With Status of p53 and MDM2 SNP309**. *JNCI: Journal of the National Cancer Institute* 2006, **98**(13):911-919.

11.	Teschendorff AE, Gao Y, Jones A, Ruebner M, Beckmann MW, Wachter DL, Fasching PA, Widschwendter M: **DNA methylation outliers in normal breast tissue identify field defects that are enriched in cancer**. *Nat Commun* 2016, **7**:10478.

12.	Teschendorff AE, Jones A, Widschwendter M: **Stochastic epigenetic outliers can define field defects in cancer**. *BMC Bioinformatics* 2016, **17**:178.

13.	Gao Y, Jones A, Fasching PA, Ruebner M, Beckmann MW, Widschwendter M, Teschendorff AE: **The integrative epigenomic-transcriptomic landscape of ER positive breast cancer**. *Clin Epigenetics* 2015, **7**:126.

14.	Gao Y, Widschwendter M, Teschendorff AE: **DNA Methylation Patterns in Normal Tissue Correlate more Strongly with Breast Cancer Status than Copy-Number Variants**. *EBioMedicine* 2018, **31**:243-252.

15.	Yang Z, Jones A, Widschwendter M, Teschendorff AE: **An integrative pan-cancer-wide analysis of epigenetic enzymes reveals universal patterns of epigenomic deregulation in cancer**. *Genome Biol* 2015, **16**(1):140.

16.	Mathe A, Wong-Brown M, Locke WJ, Stirzaker C, Braye SG, Forbes JF, Clark SJ, Avery-Kiejda KA, Scott RJ: **DNA methylation profile of triple negative breast cancer-specific genes comparing lymph node positive patients to lymph node negative patients**. *Sci Rep* 2016, **6**:33435.

17.	Carpenter JE, Clarke CL: **Biobanking Sustainability—Experiences of the Australian Breast Cancer Tissue Bank (ABCTB)**. *Biopreservation and Biobanking* 2014, **12**(6):395-401.

18.	Holm K, Staaf J, Lauss M, Aine M, Lindgren D, Bendahl P-O, Vallon-Christersson J, Barkardottir RB, Höglund M, Borg Å *et al*: **An integrated genomics analysis of epigenetic subtypes in human breast tumors links DNA methylation patterns to chromatin states in normal mammary cells**. *Breast Cancer Research* 2016, **18**(1):27.

19.	Karlsson A, Jönsson M, Lauss M, Brunnström H, Jönsson P, Borg Å, Jönsson G, Ringnér M, Planck M, Staaf J: **Genome-wide DNA Methylation Analysis of Lung Carcinoma Reveals One Neuroendocrine and Four Adenocarcinoma Epitypes Associated with Patient Outcome**. *Clinical Cancer Research* 2014, **20**(23):6127-6140.

20.	Fackler MJ, Cho S, Cope L, Gabrielson E, Visvanathan K, Wilsbach K, Meir-Levi D, Lynch CF, Marks J, Geradts J: **DNA methylation markers predict recurrence-free interval in triple-negative breast cancer**. *NPJ breast cancer* 2020, **6**(1):3.

21.	de Almeida BP, Apolónio JD, Binnie A, Castelo-Branco P: **Roadmap of DNA methylation in breast cancer identifies novel prognostic biomarkers**. *BMC Cancer* 2019, **19**(1):219.

22.	Kim DH, Binder AM, Zhou H, Jung SY: **DNA methylation patterns associated with breast cancer prognosis that are specific to tumor subtype and menopausal status**. *Frontiers in Genetics* 2023, **14**.

23.	Zarean E, Li S, Wong EM, Makalic E, Milne RL, Giles GG, McLean C, Southey MC, Dugué P-A: **Tumour DNA methylation markers associated with breast cancer survival: a replication study**. *Breast Cancer Research* 2025, **27**(1):9.
